# Anti-septin complex positive autoimmune encephalitis after severe falciparum malaria: a case report

**DOI:** 10.1186/s12936-024-05207-3

**Published:** 2024-12-11

**Authors:** Sven Kalbitz, Friederike A. Arlt, Johannes Wolf, Merle Corty, Harald Prüss, Christoph Lübbert

**Affiliations:** 1 Department of Infectious Diseases and Tropical Medicine, Hospital St. Georg, Leipzig, Germany; 2 German Centre for Neurodegenerative Diseases (DZNE) Berlin, Berlin, Germany; 3 Department of Neurology and Experimental Neurology, Charité-Universitätsmedizin Berlin, Corporate Member of Freie Universität Berlin, Humboldt-Universität Berlin, Berlin, Germany; 4 Department of Laboratory Medicine, Hospital St. Georg, Leipzig, Germany; 5 Jeffrey Modell Diagnostic and Research Centre for Primary Immunodeficiency Diseases, ImmunoDeficiency Centre Leipzig (IDCL) at Hospital St. Georg, Leipzig, Germany; 6 Clinical Immunology Laboratory Prof. Dr. Winfried Stöcker, Groß Grönau, Germany; 7 Division of Infectious Diseases and Tropical Medicine, Department of Medicine I, Leipzig University Medical Centre, Liebigstr. 20, 04103 Leipzig, Germany; 8 Interdisciplinary Centre for Infectious Diseases (ZINF), Leipzig University Medical Centre, Leipzig, Germany

**Keywords:** Septin complex, Anti-septin antibodies, Autoimmunity, Autoimmune encephalitis, Falciparum malaria, Cerebral malaria, Post malaria neurologic syndrome (PMNS), Case report

## Abstract

**Background:**

Post malaria neurologic syndrome (PMNS) is a rare complication of malaria, usually caused by *Plasmodium falciparum*. The clinical picture is highly variable and ranges from qualitative disturbances of consciousness and psychosis to damage to the peripheral nerves, usually occurring three to eight weeks after treated malaria.

**Case presentation:**

We report the case of a 54-year-old male who presented with recurrent clinical symptoms three and a half weeks after severe falciparum malaria. After ruling out recurrent malaria, autoimmune encephalitis was suspected. Corticosteroid therapy led to a rapid improvement of the clinical symptoms. The extended examinations (including cranial MRI and FDG-PET/CT) revealed no pathological findings. Routine serologic autoimmune diagnostics remained negative. However, anti-septin complex antibodies were detected in the serum in a cell-based and a tissue-based immunofluorescence assay. Twelve months after discontinuation of corticosteroid therapy, the patient was free of immunosuppressants and completely asymptomatic.

**Conclusion:**

To our knowledge, this is the first case of septin complex autoimmunity with encephalitis associated with PMNS. All physicians treating malaria patients should therefore be aware of this rare condition and consider extended autoimmune diagnostics if routine panels remain unremarkable.

## Background

Malaria is a life-threatening tropical disease and remains one of the most important global health problems [1]. According to the World Health Organization (WHO), 249 million people contracted malaria in 2022 and an estimated 608,000 died from it, 76% of whom were children under the age of five [2]. Malaria can affect any organ system, with involvement of the central nervous system (CNS) being the most feared [3]. Cerebral malaria has a mortality rate of 15–20%, and even long-term survivors often suffer from neurocognitive sequelae [4]. The diagnostic patterns range from diffuse encephalopathy with subsequent seizures to coma. Other organ dysfunctions such as kidney and liver failure usually occur in adults, while isolated cerebral malaria is more common in children. In endemic areas, almost half of children admitted to hospitals present with neurological symptoms [5]. The aetiopathogenesis of cerebral malaria is not fully understood [6]. It is a multifactorial process triggered by sequestration and adhesion of red blood cells, an immunologic response and loss of blood–brain barrier integrity [4].

Post malaria neurologic syndrome (PMNS) was first described in 1996 in patients suffering from acute confusion, psychosis or seizures within two days to two months after recovery from cerebral malaria [7]. The exact incidence is unknown due to misdiagnosis and underreporting, and there is no general definition of PMNS. In the largest prospective study conducted to date in Vietnam, the risk of developing PMNS was reported to be 1.2 per 1000 malaria cases [7]. Almost all cases occur after a severe infection with *Plasmodium falciparum*, much less frequently after an infection with *Plasmodium vivax*. According to the literature, PMNS can be divided into four categories: (1) delayed cerebellar ataxia (DCA), (2) acute inflammatory demyelinating polyneuropathy (AIDP)-like syndrome, namely Guillain-Barré syndrome, (3) acute disseminated encephalomyelitis (ADEM)-like syndrome, and (4) “classic” PMNS [8], with the last form appearing to be the most common. The most important autoantibody found to date with clinical significance in PMNS was directed against neurexin-3α. The evidence for this comes primarily from a small case series from Spain and the USA and a single case from Portugal [9, 10]. Corticosteroids have been used in several situations, but there have also been reports of self-limiting disease.

## Case presentation

We report on the case of a 54-year-old German male who was on a business trip to Chad for two weeks. In the second week of his stay abroad, symptoms began with nausea, vomiting and diarrhoea and led to an immediate return to his home country. After his arrival in Germany, the neurocognitive symptoms (misbehaviour, headaches) worsened. The patient was admitted to our infectious diseases ward one week after the onset of symptoms. We saw an awake, cognitively impaired patient (Glasgow coma scale [GCS] 13) with stable vital signs and no liver or renal dysfunction. Severe thrombocytopenia (32 Gpt/L) was conspicuous. The patient's condition deteriorated rapidly and somnolence (GCS 8) developed within 24 h, requiring intensive cardio-circulatory monitoring. *Plasmodium falciparum* was detected in blood smears with a parasite density of 14%. Antiprotozoal therapy with artesunate (2.4 mg per kg body weight IV) was initiated. After 24 h, the parasite density had decreased to less than 1% and oral follow-up therapy with artemether/lumefantrine was completed. In the further course of the disease, no more malaria parasites could be detected. Due to the severe cerebral form of falciparum malaria, a cranial MRI examination was performed, which revealed unremarkable findings. The electroencephalographic examination showed no abnormalities, and electroneurography revealed a discrete peripheral polyneuropathy. The patient was discharged as cured on day eight.

**Fig. 1:**
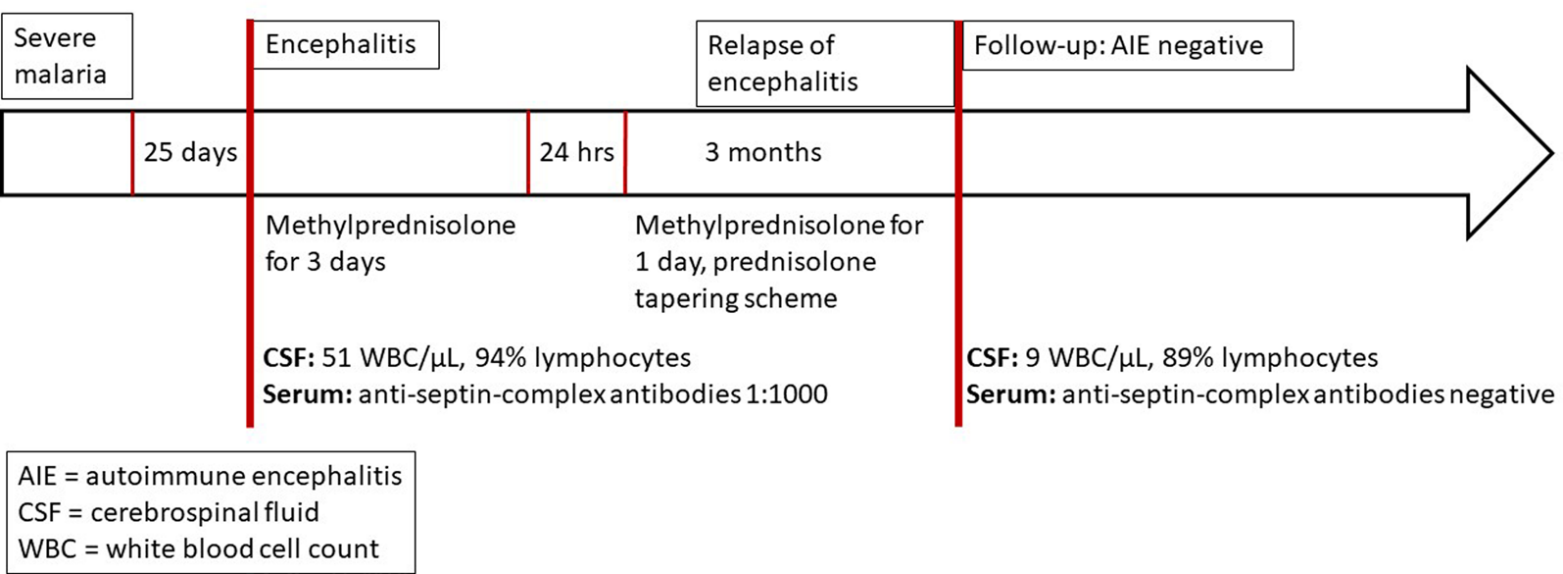
Clinical timeline

Twenty-five days after discharge, the patient developed new episodes of fever with word-finding difficulties and was readmitted. We saw a cardiocirculatory and respiratory stable patient with an encephalopathic clinical picture and motoric aphasia. A recurrence of falciparum malaria could be excluded. Cranial CT imaging showed no abnormalities, and subsequent lumbar puncture revealed a pleocytosis of 50 cells (Gpt/L) with lymphocytic predominance. Consistent with the inflammatory changes in the cerebral spinal fluid (CSF), protein was elevated to 1712 mg/L (normal range 180–430 mg/L), while lactate and glucose were normal. Multiplex PCR diagnostics for *Escherichia coli* K1, *Haemophilus influenzae, Listeria monocytogenes, Neisseria meningitidis, Streptococcus agalactiae*, *Streptococcus pneumoniae*, cytomegalovirus (CMV), enterovirus, herpes simplex virus 1 and 2 (HSV-1/2), human herpesvirus 6 (HHV-6), human parechovirus (HPeV), varicella zoster virus (VZV), West Nile virus, tick-borne encephalitis virus, *Cryptococcus neoformans*, and *Cryptococcus gattii* was negative. Bacterial cultures of the CSF remained sterile. Within 24 h, the patient's condition deteriorated rapidly and he developed apraxia and a somnolent state. Cranial MRI scans showed no abnormalities. Suspecting a neurologic autoimmune syndrome secondary to cerebral malaria, we initiated immunotherapy with methylprednisolone (250 mg IV once daily) for three days. The symptoms resolved rapidly within 24 h. However, after discontinuation of methylprednisolone, the symptoms recurred within 24 h. We therefore repeated the administration of methylprednisolone 250 mg IV as a single dose and continued the therapy with prednisolone (1 mg per kg body weight once daily). The symptoms again resolved within 24 h. Further MRI scans and FDG-PET/CT revealed no pathologic correlate. Prednisolone was tapered over two months (Fig. 1).

**Fig. 2:**
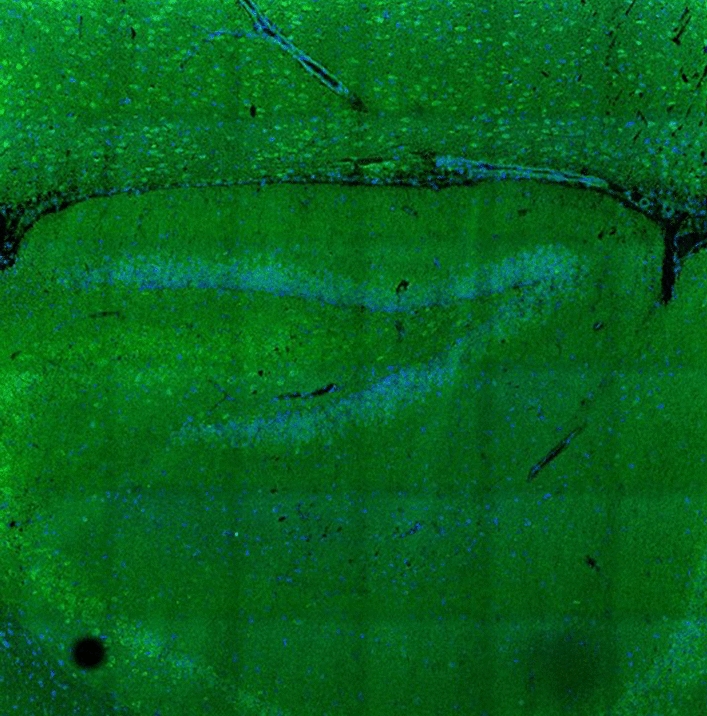
Immunofluorescence staining on central nervous tissue (rat hippocampus) with anti-septin positive patient serum (granular fluorescence of the hippocampus, inner molecular layer darker)

**Fig. 3:**
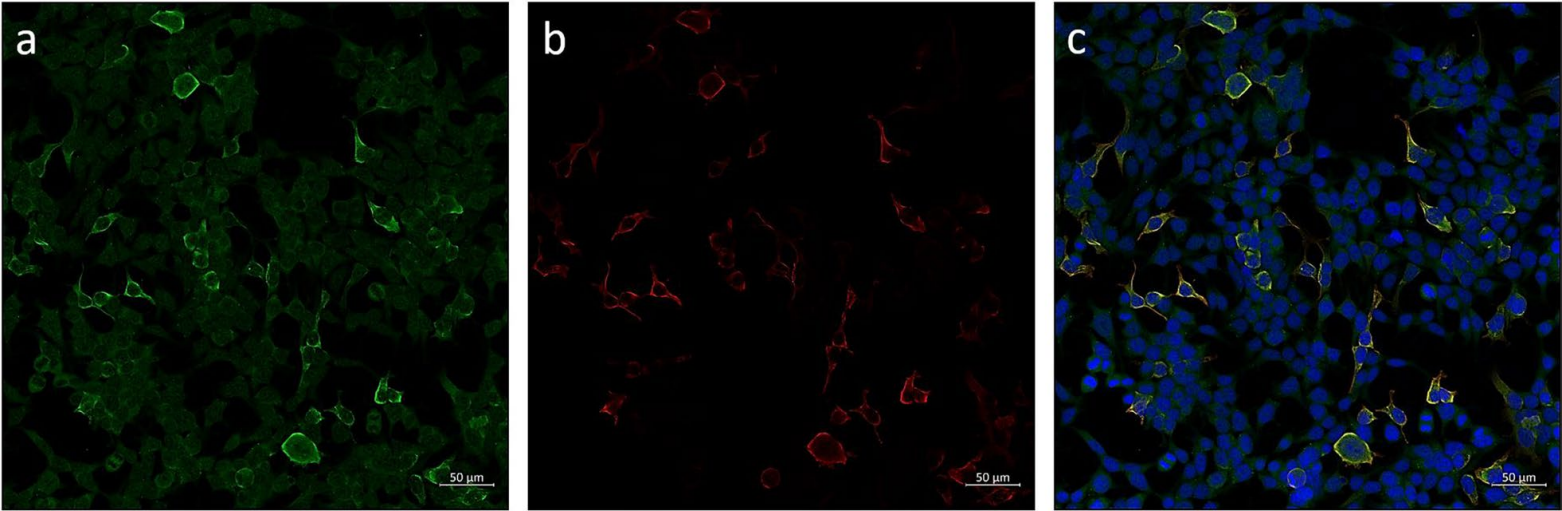
Immunofluorescence staining on a cell-based assay with septin complex transfected cells applying **a**) patient serum (diluted 1:100) followed by Alexa Fluor 488-conjugated AffinePure Goat Anti-Human IgG (Jackson Immuno Research, Baltimore Pike, USA), **b**) a polyclonal rabbit anti-SEPT3 (1:1000, Sigma-Aldrich, St.Louis, Missouri, USA) followed by Cy3-conjugated goat anti-rabbit IgG (1:200, Dianova, Hamburg, Germany) and **c**) merged, with additional staining of the cell nuclei

**Fig. 4:**
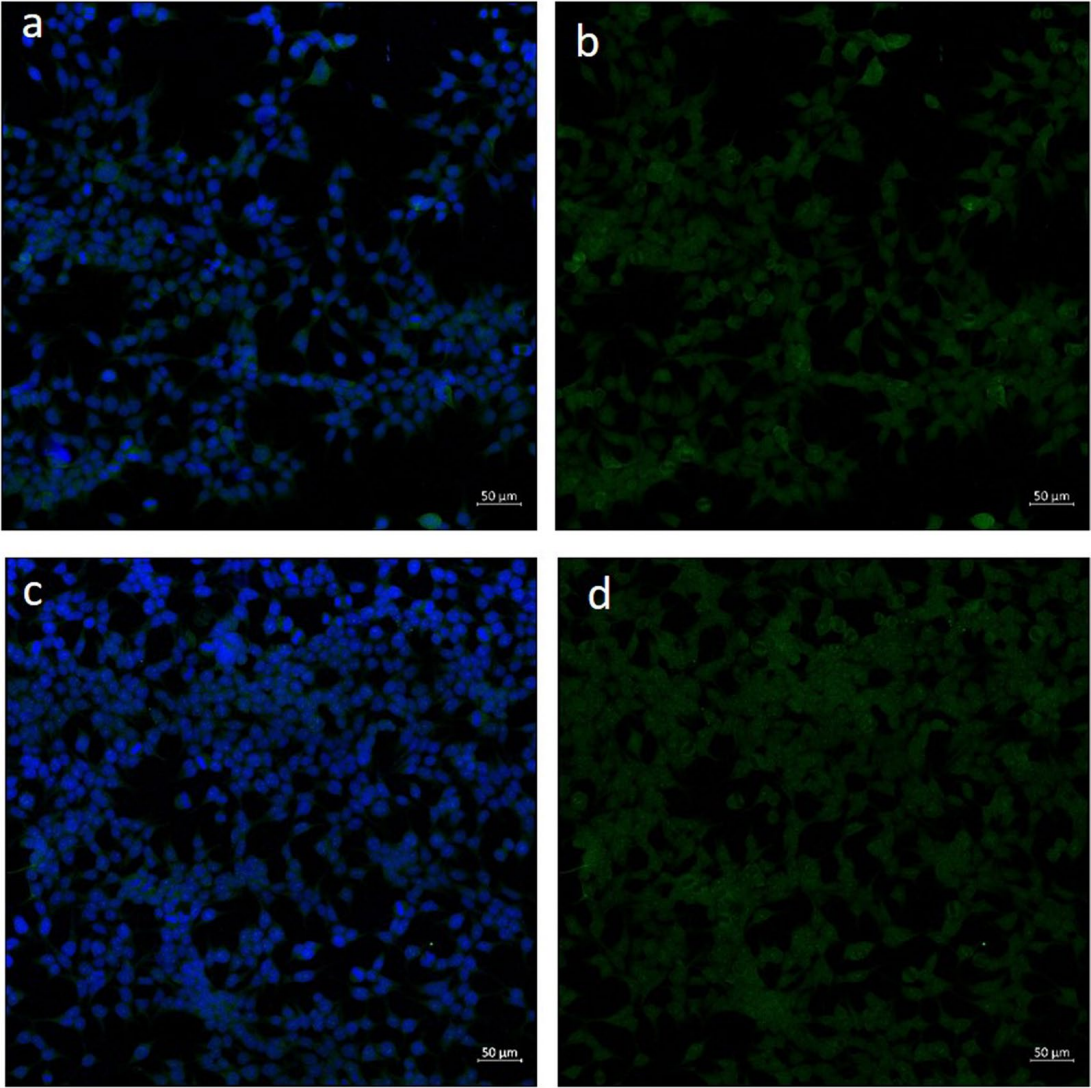
Negative results of the control examination (three months after the first examination) of the patient serum with a cell-based assay with septin complex transfected cells, **a** with and **b** without additional staining of the cell nuclei, **c** negative control with and **d** negative control without additional staining of the cell nuclei

A detailed summary of the autoantibodies measured in serum and/or CSF is given in Table 1. Autoantibodies against the septin complex (septin-3/5/6/7/11) in serum samples were detected with a tissue-based assay (rat hippocampus, titre 1: 100) and with a recombinant cell-based assay (titre 1:1000) (Figs. 2 and 3). One month after termination of prednisolone therapy, the patient was clinically completely unremarkable and had no neurological deficits. We carried out a CSF puncture check and renewed autoimmune diagnostics. A regression of the CSF cell count to 9 (Gpt/L) was documented. The anti-septin-complex antibodies were now negative, as a subsequent cell-based test demonstrated (Fig. 4). Twelve months after discontinuation of prednisolone therapy, the patient was free of immunosuppressants and completely asymptomatic.

**Table 1:** Autoimmune diagnostic work-up

Autoantibodies	Method	Result	Reference	Unit
Anti-nuclear antibodies (ANA)	IFA	1:160	< 1:80	n/a
ANA pattern	IFA	AC-25		n/a
Extractable nuclear antigen screening	FIA	0.1	< 1	ratio
Proteinase 3	FIA	< 0.2	< 2	IU/ml
Myeloperoxidase	FIA	< 0.2	< 3.5	IU/ml
Hu	LB/IFA	Negative	negative	n/a
Ri	LB/IFA	Negative	negative	n/a
Yo	LB/IFA	Negative	Negative	n/a
ANNA-3	LB/IFA	Negative	Negative	n/a
Tr/DNER	LB/IFA	Negative	Negative	n/a
GAD65	LB/IFA	Negative	Negative	n/a
Amphiphysin	LB/IFA	Negative	Negative	n/a
Ma2/Ta	LB/IFA	Negative	Negative	n/a
Sox1	LB/IFA	Negative	Negative	n/a
Zic4	LB/IFA	Negative	Negative	n/a
Recoverin	LB/IFA	Negative	Negative	n/a
Titin	LB/IFA	Negative	Negative	n/a
Myelin	IFA	**1:100**	< 1:100	n/a
Aquaporin	IFA	Negative	< 10	n/a
MOG	IFA	Negative	< 10	n/a
Glutamate receptor type NMDA in serum	CBA/IFA	Negative	< 10	n/a
Glutamate receptor type NMDA in CSF	CBA/IFA	Negative	< 1	n/a
Glutamate receptor type AMPA1 and 2	CBA/IFA	Negative	< 10	n/a
GABA-b-Receptor	CBA/IFA	Negative	< 10	n/a
LGI1	CBA/IFA	Negative	< 10	n/a
CASPR2	CBA/IFA	Negative	< 10	n/a
IgLON5	CBA/IFA	Negative	< 10	n/a
DPPX	CBA/IFA	Negative	< 10	n/a
mGluR5	IFA	Negative	< 10	n/a
Glycin receptor	IFA	Negative	< 10	n/a
ITPR1	IFA	Negative	< 10	n/a
Neurochondrin	IFA	Negative	< 10	n/a
Contactin 1	IFA	Negative	< 10	n/a
Neurofascin 155	IFA	Negative	< 10	n/a
Neurofascin 185	IFA	Negative	< 10	n/a
Neurexin-3α	CBA	Negative	< 100	n/a
Septin complex (septin-3/5/6/7/11) antibodies in serum	CBA/IFA	**1:1000**	< 1:10	n/a
Septin complex antibodies in CSF	CBA/IFA	Negative	Negative	n/a

*CBA* Cell-based assay, *CSF* Cerebrospinal fluid, *FIA* Fluorescence immune assay, *IFA* Immunofluorescence assay, *n/a* Not applicable, *LB* Line blot

## Discussion and conclusions

We report a case of PMNS which presented as autoantibody-associated autoimmune encephalitis and clearly responded to immunotherapy with corticosteroids. To our knowledge, this is the first case of autoimmune encephalitis after severe cerebral malaria in which anti-septin complex antibodies were positive. Due to the absence of MRI findings, we consider this case to be a “classic” PMNS. In particular, the foudroyant course shows that PMNS is an acute auto-inflammatory process that requires consistent immunosuppressive therapy. This is underlined by the prompt relapse that occurred after discontinuation of methylprednisolone therapy. Therefore, early initiation of anti-inflammatory therapy is crucial in these patients and should not be delayed due to the uncertainty of an autoimmune diagnosis. Sahuguet et al*.* [11] published a clinically similar case of PMNS, in which specific antibodies against the voltage-gated potassium channel (VGKC) were detected, possibly triggering autoimmune encephalitis leading to PMNS. Antibodies against VGKC were not found in our patient. Comprehensive serological analyses by British neuroscientists suggest that these antibodies should no longer be classified as neuronal surface antibodies if only VGKC is positive and LGI1 and CASPR2 are negative [12]. Consequently, they have no pathogenic potential and do not in themselves support the use of immunotherapies. Autoantibodies against neurexin-3α, for which the pathophysiological significance is much clearer, were also not detected in our patient.

Septins are a family of GTP-binding proteins. They are ubiquitously expressed including neuronal tissue and play a crucial role in the organization of the cytoskeleton and in cell division as well as in the organization of endo- and exocytosis at synaptic terminals [13]. Antibodies against septin-3 have been described in patients with cerebral ataxia suffering from various malignancies and may represent a new target for paraneoplastic neurological syndromes [14]. Anti-septin-5 antibodies are associated with autoimmune encephalitis and cerebral ataxia [15–17]. Septin genes are not expressed in protozoa [18]. Direct antigen presentation by *Plasmodium falciparum*, which could have triggered autoimmunity, is therefore unlikely. No septin complex antibodies could be detected in the CSF of our patient, which does not rule out the possibility of peripheral antibody formation and subsequent migration through a disrupted blood–brain barrier. Whether septin complex antibodies were already present in the serum at the time of infection or whether they developed secondary to inflammation could unfortunately not be determined. In principle, molecular mimicry with the formation of cross-reactive antibodies seems plausible, comparable to other autoimmune syndromes. However, whether infections with protozoa per se can trigger molecular mimicry remains unclear. Furthermore, the pathogenic effect of septin complex antibodies is still unclear, while binding to living cells and in-vitro effects on neuronal cultures have already been demonstrated for septin-5-IgG and septin-7-IgG antibodies [17]. Since no septin complex antibodies were detected in the CSF of our patient, reasonable doubts remain about their pathogenic role. However, we could not identify any other competing biomarker for autoimmune encephalitis.

The diagnosis of autoimmune encephalitis is based on a comprehensive evaluation of the clinical picture, CSF and radiological findings, and electroencephalogram (EEG) results [19]. Autoantibody status and clinical response are not considered in the early diagnostic phase. All the diagnostic criteria mentioned above were fully met in the present case. As this is the first description of PMNS in which anti-septin complex antibodies have been detected, extrapolation of data from other cases of “classic” autoimmune encephalitis may be somewhat premature. However, the presence of autoantibodies in the CSF is not a mandatory requirement for the diagnosis of autoimmune encephalitis [19]. So far, this question has been discussed mainly in the context of NMDA receptor-positive autoimmune encephalitis.

In summary, we conclude that secondary autoimmune encephalitis after severe falciparum malaria may be mediated by anti-septin complex antibodies, which was previously unknown. All physicians treating malaria patients should, therefore, be aware of this rare condition and consider extended autoimmune diagnostics if routine panels remain unremarkable.

## Data Availability

No datasets were generated or analysed during the current study.

## Abbreviations

ADEM: Acute disseminated encephalomyelitis
AIDP: Acute inflammatory demyelinating polyneuropathy
ANA: Anti-nuclear antibodies
CBA: Cell-based assay
CMV: Human cytomegalovirus
CNS: Central nervous system
CSF: Cerebrospinal fluid
CT: Computed tomography
DCA: Delayed cerebellar ataxia
EEG: Electroencephalogram
FDG-PET/CT: ^18^Fluoro-deoxyglucose positron emission tomography/computed tomography
GCS: Glasgow coma scale
Gpt/L: Giga-parts per litre
GTP: Guanosine triphosphate
HHV: Human herpes virus
HPeV: Human parechovirus
HSV: Herpes simplex virus
IFA: Immunofluorescence assay
IgG: Immunoglobulin G
IV: Intravenous
kg: Kilogram
mg: Milligram
MRI: Magnetic resonance imaging
n/a: Not applicable
PCR: Polymerase-chain reaction
PMNS: Post-malaria neurologic syndrome
VGKC: Voltage-gated potassium channel
VZV: Varicella zoster virus
WHO: World Health Organization
